# Depressive Symptoms and Behavior-Related Risk Factors, Italian Population-Based Surveillance System, 2013

**DOI:** 10.5888/pcd12.150154

**Published:** 2015-10-29

**Authors:** Antonella Gigantesco, Gianluigi Ferrante, Sandro Baldissera, Maria Masocco, on behalf of the PASSI coordinating group

**Affiliations:** Author Affiliations: Antonella Gigantesco, Sandro Baldissera, Maria Masocco, National Institute of Public Health, Rome, Italy.

## Abstract

**Introduction:**

Depression may increase the likelihood of adopting behaviors risky to health. Population studies investigating the association between depressive symptoms and behavior-related risk factors are lacking in Italy. The aim of this study was to estimate the prevalence of various self-reported behavior-related risk factors and to study their associations with current depressive symptoms in the Italian adult general population.

**Methods:**

Data collected in 2013 from people aged 18 to 69 years participating in the Italian behavioral risk factor surveillance system were used for the analysis. Indicators of no leisure-time physical activity, obesity, cigarette smoking, and excessive alcohol consumption were investigated. Depressive symptoms were explored through the Patient Health Questionnaire-2.

**Results:**

In the survey sample of 39,463 participants, 34.4% of adults engaged in no leisure-time physical activity, 26.2% were cigarette smokers, 11.5% were excessive alcohol consumers, and 10.3% were obese. The prevalence of depressive symptoms was 6.2%. People with depressive symptoms were more likely to be physically inactive (adjusted prevalence ratio [APR], 1.13), cigarette smokers (APR, 1.34), obese (APR, 1.27) and excessive alcohol consumers (APR, 1.43) than those without depressive symptoms.

**Conclusion:**

The contribution of this study to the existing evidence lies not just in confirming the association between depression and behavior-related risk factors in the Italian context but also in suggesting that programs for simultaneously improving people’s mental and physical health should be developed and implemented.

## Introduction

Depression affects approximately 350 million people worldwide (1) and has major public health implications. It is the fourth leading cause of worldwide disease burden, accounting for 12% of all years lived with disability (2). Depression has severe consequences in terms of economic cost, which has been estimated for the year 2000 at $83.1 billion in the United States (3). In patients with comorbid physical illness, depression is associated with higher medical costs, increased physical symptom burden, greater impairment in social functioning, and poorer self-care and adherence to medical treatment (4).

Depression worsens the course and outcome of many common chronic conditions, such as cardiovascular disease (5). Depression may not only compound existing physical disorders but it can also increase the risk for other comorbidities. Population-based studies have investigated the relationship between depression and behavioral risk factors such as tobacco use, alcohol use, binge drinking, physical inactivity, and certain eating habits (6,7). These studies showed that in people with depression, those behaviors tend to occur more frequently than in the general population. There is also evidence that these same behavioral factors can lead to depression (8).

Making use of data from the Italian behavioral risk factor surveillance system PASSI (Progressi delle Aziende Sanitarie per la Salute in Italia), we describe how different behavior-related risk factors are distributed in the Italian population and examine whether their occurrence is associated with the presence of depressive symptoms. The behaviors investigated were cigarette smoking, physical inactivity, excessive alcohol consumption, and obesity, which are major risk factors for chronic diseases and priority targets for international health promotion programs. Population studies investigating the association between depressive symptoms and behavior-related risk factors are lacking in Italy, and this work aims to close the knowledge gap.

## Methods

### Population data source

The sample for this study was extracted from the Italian adult population aged 18 to 69 years, comprising 40,289,221 people on January 1, 2013. In Italy, universal health care is administered by the regions within the framework of the National Health System. Each of the 20 Italian regions comprises between 1 and 22 local health units (LHUs), which provide preventive and curative services for populations ranging from 40,000 to over 1 million.

The unit of data collection in PASSI is the LHU. The target population consists of all people aged 18 to 69 years residing in the LHU area. The survey (eligible) population comprises residents who have a telephone number available and are capable of being interviewed (9). In each LHU, a random sample is drawn monthly from the LHU’s enrollment list of residents, stratified by sex and age (18–34, 35–49, and 50–69) proportionally to the size of the respective stratum in the general population. Both landline and cellular telephones are used for the interviews.

### Data collection

Specially trained personnel from the public health departments of each LHU administer telephone interviews through a standardized questionnaire, gathering information on physical and mental health, behavior-related risk factors, and sociodemographic characteristics. Interviews collected during a calendar year are aggregated in an annual data set. The LHUs’ data are merged and analyzed to obtain regional and national estimates. More details on the data system have been described (9).

The depressive symptoms screening module used by PASSI is the Patient Health Questionnaire 2 (PHQ-2) (10). With reference to an accepted gold standard, the Structured Clinical Interview for Diagnostic and Statistical Manual of Mental Disorders, fourth edition, the PHQ-2 showed a sensitivity of 87% and a specificity of 78% for major depressive disorder (11). In population surveys, because of the low prevalence of major depression, the use of PHQ-2 leads to an overestimation of the disease, up to 2 to 3 times that obtained with more specific instruments. In a study of the Italian general population, the PHQ-2 showed good sensitivity (98%) and specificity (90%) but low positive predictive value (37%) compared with the PHQ-8 (12), a screening instrument widely used in population-based surveys (13) and more accurate than the PHQ-2 in predicting major depressive disorder.

For the purposes of the survey, PASSI chose the format and response categories adapted by the original developers to the United States’ Behavioral Risk Factor Surveillance System (14). In this adaptation, the first question asks how many days in the past 2 weeks the person experienced little interest or pleasure in doing things, and the second asks the number of days in which he or she felt down, depressed, or without hope; in both cases, the response is between 0 and 14 (15). The number of days is recoded on a point scale between 0 and 3 for each of the 2 questions: 0 to 1 day is coded as 0, 2 to 6 days as 1, 7 to 11 days as 2, and 12 to 14 days as 3; the points are then summed. Those with a summed score of 3 or more are considered to have depressive symptoms (case definition); this value corresponds to that demonstrated to be optimal for major depression screening purposes (10).

Respondents were considered to be obese if their body mass index (BMI), calculated from their self-reported weight and height (kg/m^2^), was 30 or higher (16). Cigarette smokers were defined as those who report having smoked at least 100 cigarettes in their lifetime and being current smokers on every day or some days (17).

Leisure-time physical activity is explored by asking interviewees if in the 30 days before the interview they have engaged in moderate physical activity (vacuuming, gardening, brisk walking, or bicycling) or vigorous physical activity (running, aerobics, or heavy yard work), without considering working time; additionally, for both levels of physical activity, they were asked for how many days in a week and for how many minutes in those days they exercised, on average. The products of the latter 2 values were obtained for each level of physical activity; then an overall measure of weekly physical activity was obtained by doubling the minutes of weekly vigorous physical activity and adding this value to the minutes of weekly moderate physical activity, because 1 minute of vigorous physical activity is assumed to be equivalent to 2 minutes of moderate physical activity (18). Daily physical activity bouts of less than 10 minutes’ duration were not included in the calculation. Respondents were considered to engage in no leisure-time physical activity if the indicator of weekly leisure-time physical activity, as defined above, was equal to 0.

Men were considered to be excessive alcohol consumers if they drank more than 2 alcoholic units (AUs) per day on average or if they reported having 5 or more AUs during a single occasion in the 30 days before the interview. For women, the threshold was more than 1 AU per day on average or 4 or more AUs during a single occasion. One AU corresponds to a glass of wine (125 mL), a can of beer (330 mL), or 1 shot glass of spirits (40 mL) (20).

The PASSI questionnaire collects information on sociodemographic characteristics. Those included in the analysis were sex, age (18–24, 25–34, 35–49, or 50–69 y), completed education level (none or elementary school, junior high school, high school, or university), marital status (married, single, widowed, or divorced), employment status (continuously employed, temporarily employed, or unemployed), economic difficulties (many, some, or no difficulties in getting to the end of the month with the available income), and geographic area of residence (North, Center, or South and major islands, according to the criteria of the Italian National Institute of Statistics). The presence of at least 1 chronic disease was defined as having ever received by a physician a diagnosis of a condition among those specified in the questionnaire: diabetes, renal insufficiency, asthma, chronic respiratory disease, chronic liver disease, cancer, coronary heart disease or other cardiovascular disease, or cerebral stroke or brain ischemia.

### Analysis

Data from 2013 were used to perform different types of analyses. First, we described the distribution of sociodemographic characteristics and chronic medical conditions and the prevalence of obesity, current cigarette smoking, physical inactivity, and excessive alcohol consumption in each category of the study population. Second, we estimated the occurrence of depressive symptoms, overall and by sociodemographic characteristics and chronic medical conditions, reporting crude prevalence ratios among categories. Third, we described the occurrence of the 4 behavior-related risk factors among people with and without depressive symptoms. For each of these risk factors, bivariate and multivariate analyses were performed to assess the relationship between the single risk factor (dependent variable) and depressive symptoms (independent variable), controlling for sociodemographic characteristics and presence of chronic disease.

Complex survey design analyses, using the Taylor series method for variance estimation, were conducted in Stata 12 software (StataCorp LP). Prevalence estimates were weighted, assigning each record a probability weight equal to the inverse of the sampling fraction in each LHU stratum. Prevalence ratios (adjusted and unadjusted) were calculated using the Poisson regression with robust variance and hypothesis testing (19).

## Results

In 2013, 130 out of 143 Italian LHUs participated in PASSI. The population aged 18 to 69 years resident in the participating LHUs corresponded to 93% of the Italian population of the same age. The total number of interviews collected in 2013 was 40,502. Response rate, calculated according to the American Association for Public Opinion Research RR4 standard, was 84%. Data on depressive symptoms were available for 39,463 interviews (97.4%); in this sample, item nonresponse for the behavior-related risk factors was negligible (0.2% for excessive alcohol consumption and <0.1% for the other factors).

Most participants were married (58.7%), had a high level of education (high school or university: 62.4%), and had a stable job (53.5%) (Table 1). One out of 6 (17.2%) claimed considerable economic difficulties, and 42.4% and 40.4% reported some difficulties or no difficulties at all, respectively. At least 1 chronic disease was reported by 18.1% of interviewees. One out of 10 (10.3%) was obese, more than one-fourth were current cigarette smokers (26.2%), and more than one-third engaged in no leisure-time physical activity (34.4%). Excessive alcohol consumption was reported by 11.5% of interviewees. At least 1 risk factor was present in about 60% of the population; more precisely, 1 factor in 40.1%, 2 in 16.5%, 3 in 2.7%, and 4 in 0.2%.

**Table 1:** Distribution of Sociodemographic Characteristics and Chronic Medical Conditions in the Italian Adult Population (18–69 Years) and Prevalence of Behavior-Related Risk Factors, PASSI, 2013 (n = 39,463)

Characteristic	Distribution, %	Behavior-Related Risk Factors (%)			
		Obesity^a^	Current Smoking^b^	No Leisure-Time Physical Activity^c^	Excessive Alcohol Consumption^d^
**Overall**	100	10.3	26.2	34.4	11.5
**Sex**					
Male	49.5	11.3	30.2	34.8	15.9
Female	50.5	9.4	22.1	33.9	7.3
**Age group, y**					
18–24	11.3	3.4	27.1	26.5	15.5
25–34	16.9	5.8	30.2	29.7	13.2
35–49	34.9	8.9	28.0	35.5	10.3
50–69	36.9	15.9	22.3	37.8	10.7
**Marital status**					
Married	58.7	12.4	22.5	37.1	9.5
Single	33.6	6.2	31.0	29.3	15.0
Widowed	2.3	16.5	24.2	39.6	9.1
Divorced	5.4	11.0	36.5	33.5	12.0
**Completed education level**					
University	15.8	5.8	19.5	25.6	11.7
High school	46.6	7.9	26.4	31.8	11.5
Junior high school	29.6	13.6	30.3	40.0	11.7
None or elementary school	8.0	21.7	22.6	45.5	10.0
**Employment status**					
Continuously employed	53.5	8.8	27.9	33.6	12.4
Temporarily employed	7.7	8.3	31.6	37.3	13.5
Unemployed	38.8	12.8	22.6	34.8	9.8
**Economic difficulties^e^**					
None	40.4	7.6	21.9	27.7	13.1
Some	42.4	10.8	26.9	36.2	10.5
Many	17.2	15.6	34.4	45.5	10.2
**Geographic area of residence**					
South and major islands	37.9	11.8	26.7	43.2	8.2
Center	22.0	8.9	28.4	31.6	11.1
North	40.2	9.7	24.5	27.5	14.7
**At least 1 chronic disease**					
No	81.9	8.5	26.3	33.2	11.5
Yes	18.1	18.5	25.5	39.3	11.5

Abbreviation: PASSI, the Italian behavioral risk factor surveillance system (Progressi delle Aziende Sanitarie per la Salute in Italia).

a Body mass index (BMI) ≥30 kg/m^2^.

b Reporting having smoked at least 100 cigarettes in their lifetime and being current smokers on every day or some days.

c No moderate (vacuuming, gardening, brisk walking, or bicycling) or vigorous (running, aerobics, heavy yard work) physical activity for at least 10 minutes per week in the previous 30 days. Daily physical activity bouts of less than 10 minutes’ duration were not included in the calculation.

d For men, more than 2 alcoholic units (AUs) per day on average or 5 or more AUs during a single occasion in the previous 30 days. For women, more than 1 AU per day on average or 4 or more AUs during a single occasion in the previous 30 days. One AU corresponds to a glass of wine (125 mL), a can of beer (330 mL), or 1 shot glass of spirits (40 mL).

e Question: “With the financial resources you have at your disposition, either from your income or from the family, how do you get to the end of the month?” Answers: “easily or very easily/with some difficulties/with much difficulties.”

A total of 6.2% (95% confidence interval [CI], 5.8%–6.4%) met the case definition of presence of depressive symptoms. This percentage was significantly higher among women, older people (those aged 50 to 69 vs those aged 18 to 24), widowed and divorced persons compared with married persons, those with many or some economic difficulties compared with those without difficulties, people with a lower educational level (those with none or elementary school education and those with junior high school education compared with those with a university degree), unemployed or temporarily employed people compared with those continuously employed, and those with at least 1 chronic disease compared with those without chronic disease (Table 2).

**Table 2:** Prevalence of Depressive Symptoms, by Sociodemographic Characteristics and Chronic Medical Conditions, and Crude Prevalence Ratios Among Categories in the Italian Adult Population (18–69 years), PASSI, 2013 (n = 39,463)

Characteristic	Prevalence of Depressive Symptoms, %	Crude Prevalence Ratio^a^	*P* Value
**Overall**	6.2	NA	
**Sex**			
Male	4.5	1 [Reference]	
Female	7.8	1.75	<.001
**Age group, y**			
18–24	4.9	1 [Reference]	
25–34	4.4	0.89	.32
35–49	5.6	1.14	.17
50–69	7.9	1.62	<.001
**Marital status**			
Married	5.7	1 [Reference]	
Single	5.7	0.99	.85
Widowed	12.0	2.08	<.001
Divorced	10.9	1.90	<.001
**Educational level**			
University	4.0	1 [Reference]	
High school	5.3	1.32	.002
Junior high school	7.3	1.81	<.001
None/elementary school	11.1	2.74	<.001
**Employment status**			
Continuously employed	4.5	1 [Reference]	
Temporary employed	6.1	1.37	.007
Unemployed	8.4	1.87	<.001
**Economic difficulties^b^**			
None	3.5	1 [Reference]	
Some	5.8	1.66	<.001
Many	13.2	3.76	<.001
**Geographic area of residence**			
South and major islands	5.8	1 [Reference]	
Center	6.6	1.15	.03
North	6.3	1.09	.18
**At least 1 chronic disease^c^**			
No	4.7	1 [Reference]	
Yes	12.9	2.76	<.001

Abbreviations: NA, not applicable: PASSI, the Italian behavioral risk factor surveillance system (Progressi delle Aziende Sanitarie per la Salute in Italia).

a Calculated through Poisson regression.

b Question: “With the financial resources you have at your disposition, either from your income or from the family, how do you get to the end of the month?” Answers: “easily or very easily/with some difficulties/with much difficulties.”

c Diabetes, renal insufficiency, asthma, chronic respiratory disease, chronic liver disease, cancer, coronary heart disease or other cardiovascular disease, or cerebral stroke or brain ischemia.

The Figure shows the occurrence of the 4 behavior-related risk factors in people with and without depressive symptoms.

**Figure:**
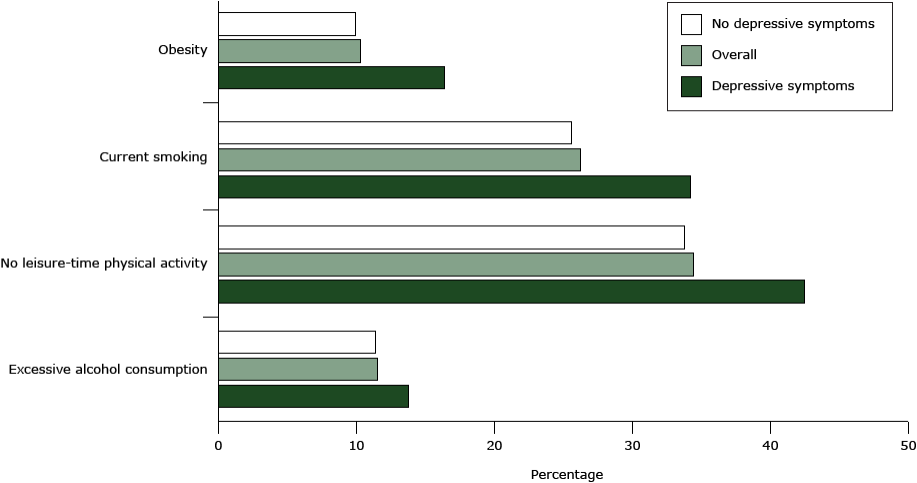
Occurrence of behavior-related risk factors in the Italian adult population (18–69 y), overall and in people with and without depressive symptoms from the Italian behavioral risk factor surveillance system PASSI (Progressi delle Aziende Sanitarie per la Salute in Italia), 2013 (n = 39,463). Obesity was defined as a body mass index (BMI) of 30 kg/m^2^ or greater. Current smoking was defined as reporting having smoked at least 100 cigarettes in their lifetime and being current smokers on every day or some days. No leisure-time physical activity was defined as no moderate (vacuuming, gardening, brisk walking, or bicycling) or vigorous (running, aerobics, or heavy yard work) physical activity for at least 10 minutes per week in the previous 30 days. Daily physical activity bouts of less than 10 minutes’ duration were not included in the calculation. Excessive alcohol consumption was defined for men as more than 2 alcoholic units (AUs) per day on average or 5 or more AUs during a single occasion in the previous 30 days; for women, it was defined as more than 1 AU per day on average or 4 or more AUs during a single occasion in the previous 30 days. One AU corresponds to a glass of wine (125 mL), a can of beer (330 mL), or 1 shot glass of spirits (40 mL). **Table:** 

Risk Factor	No Depressive Symptoms	Overall	Depressive Symptoms
Excessive alcohol consumption	11.4	11.5	13.8
No leisure-time physical activity	33.8	34.4	42.5
Current smoking	25.6	26.2	34.2
Obesity	9.9	10.3	16.4

A significant association between depressive symptoms and behavior-related risk factors was confirmed after adjusting for sociodemographic characteristics and medical conditions: people with depressive symptoms were significantly more likely than those without depressive symptoms to engage in no leisure-time physical activity (adjusted prevalence ratio [APR], 1.13; 95% CI, 1.04–1.22), to be obese (APR, 1.27; 95% CI, 1.08–1.48), to be current smokers (APR, 1.34; 95% CI, 1.21–1.48), and to be excessive alcohol consumers (APR, 1.43; 95% CI, 1.17–1.74) (Table 3). No effect modification by sex was observed.

**Table 3:** Unadjusted and Adjusted Prevalence Ratio of Behavior-Related Risk Factors Between People With and Without Depressive Symptoms in the Italian Adult Population (18–69 years), PASSI, 2013 (n = 39,463)

Risk Factor	Occurrence of Depressive Symptoms, % (95% CI)		Unadjusted Prevalence Ratio^a^ (*P* Value)	Adjusted^v^ Prevalence Ratio^a^ (*P* Value)
	No	Yes		
**Obesity^c^**	9.9 (9.6–10.3)	16.4 (14.6–18.4)	1.65 (<.001)	1.27 (.003)
**Current smoking^d^**	25.6 (25.1–26.2)	34.2 (31.8–36.7)	1.33 (<.001)	1.34 (<.001)
**No leisure-time physical activity^e^**	33.8 (33.2–34.4)	42.5 (40.0–45.0)	1.26 (<.001)	1.13 (.003)
**Excessive alcohol consumption^f^**	11.4 (11.0–11.7)	13.8 (12.1–15.6)	1.21 (.005)	1.43 (<.001)

Abbreviations: CI, confidence interval; PASSI, Italian behavioral risk factor surveillance system (Progressi delle Aziende Sanitarie per la Salute in Italia).

a Reference group is people without depressive symptoms.

b Adjusted by sex, age, marital status, educational level, economic difficulties, employment status, geographic area of residence, and occurrence of chronic disease.

c Body mass index ≥30 kg/m^2^.

d Reporting having smoked at least 100 cigarettes in their lifetime and being current smokers on every day or some days.

e No moderate (vacuuming, gardening, brisk, walking, or bicycling) or vigorous (running, aerobics, heavy yard work) physical activity for at least 10 min per week in the previous 30 days. Daily physical activity bouts of less than 10 minutes’ duration were not included in the calculation.

f For men, more than 2 alcoholic units (AUs) per day on average or 5 or more AUs during a single occasion in the previous 30 days. For women, more than 1 AU per day on average or 4 or more AUs during a single occasion in the previous 30 days. One AU corresponds to a glass of wine (125 mL), a can of beer (330 mL), or 1 shot glass of spirits (40 mL).

## Discussion

To the best of our knowledge, this is the first study in the Italian context that examines the relationships between behavior-related risk factors and current depressive symptoms in a nationally representative sample. We found that depressive symptoms were associated with 4 major behavior-related risk factors, even after adjusting for possible confounders; this finding is consistent with reports from other countries, such as Ireland (6), France (7), and the United States (21).

In 2013, approximately 43% of Italian adults who reported depressive symptoms engaged in no leisure-time physical activity. This is a considerable problem for people with depressive symptoms, because physical exercise is an effective treatment for people with mild to moderate depression. Summarizing the available evidence, National Institute for Health and Care Excellence guidelines recommend structured supervised exercise programs 3 times a week (45–60 minutes) over 10 to 14 weeks as a low-intensity Step 2 intervention for mild to moderate depression (22).

About 34% of adults with depressive symptoms were cigarette smokers. There is an association between depression and smoking (6,7,20,21).

The percentage of excessive alcohol consumers among subjects with depressive symptoms was 13.8%. We found an association between depressive symptoms and excessive alcohol consumption that, notably, did not differ by sex. This finding diverges from previous research in other countries, which suggests that the association between depression and problem alcohol use is stronger in women than in men (21). We have no ready explanation for this discrepancy; however, an influence of the socio-cultural context could be reasonably hypothesized. The prevalence of excessive alcohol consumption we observed was substantially lower than that of other countries, both in the general population and in people with depressive symptoms (20). A low prevalence such as this could level off possible differences between sexes.

In recent studies, obesity was found to increase the risk of depression, and depression was found to be predictive of developing obesity (23). The relationships between the multiple factors here considered (depressive symptoms, behaviors, sociodemographic characteristics, and chronic conditions) are complex and bidirectional. Trying to disentangle them and to infer causal relations would require appropriate study designs. That was not an aim of our study, which was based on cross-sectional survey data.

However, also finding solid associations between these factors has implications for public health and clinical care. We estimate that in Italy each year hundreds of thousands of people who report depressive symptoms also have behavior-related risk factors. Independently of whether these factors predate or follow depressive symptoms, their coexistence may potentially initiate or intensify a dangerous spiral of mutual enhancement. Moreover, both depressive symptoms and behavior-related risk factors can cause or aggravate chronic medical conditions, which in turn are capable of fostering the onset of depressive symptoms, further worsening the health status of the affected persons.

Therefore, future intervention strategies for promoting healthier behaviors and preventing chronic diseases should consider the interconnected nature of mental health, physical disorders, and behavior patterns. Programs for screening and treatment of depression should be delivered in primary care. This can be accomplished through enhancing the role of the primary care physician in the management of depression by means of specific training and supervision, while providing the possibility of referral to dedicated mental health specialists when needed. At the same time, integrated secondary public health services are needed to address both depression and multiple risk factors. Some of the services recently proposed in the United Kingdom for integration of medical and mental health services should be considered in Italy. In the West Midlands, for example, Increasing Access to Psychological Therapy services and Health Trainer services were recently introduced; these new public health services jointly work to offer people treatment for depression and anxiety and to support them in considering and modifying their health-related behaviors (24).

There are some limitations to our study. Data collected by PASSI may be affected by various types of bias:

1. Unit nonresponse. Because nonrespondents often differ from respondents in the characteristics under consideration, nonresponse can lead to inaccurate measures of the indicators. There is no general consensus about which value of response rate can be judged acceptable, because the alteration of the estimates depends on many factors, such as the design of the survey, the examined population, the reasons for nonresponse, the explored variables, and others. However, response rates greater than 80%, like that of PASSI, are usually considered good (25). Moreover, extracting the sample from LHUs’ lists of residents and using both landline and cellular telephones for the interviews, PASSI achieves a wider coverage than other surveys (eg, random digit dialing).
2. Item nonresponse. People who refuse to respond about sensitive issues, like mental health, may differ in regard to the studied characteristics, and this may alter the estimates. In our study, information on depressive symptoms was missing in only 2.5% of collected interviews, and we estimate that even a much higher occurrence of depressive symptoms in this small subgroup of item nonrespondents would not change substantially the observed estimates and associations.
3. Self-reporting. As with any health interview survey, PASSI data (except for the main demographic characteristics: sex, age, and residence) are self-reported. Consequently, the answers can be influenced by various biases. Specifically, people may be less likely to report symptoms of depression because of the stigma surrounding mental illness, leading to an underestimation of their prevalence.
4. Measuring instruments limits. The diagnosis of major depression requires adequate measuring instruments and must be confirmed by a physician with a proper follow-up. The PHQ-2 module adopted by PASSI can detect the people at risk for depression, but substantially overestimates the prevalence of the disease in general population surveys, as mentioned; conversely, the associations with other important variables are weakened (12). Consequently, the relationships with the risk factors we observed may have been stronger if we had used a more accurate indicator of depression.

Finding associations between behavior-related risk factors and poor mental health is not a new concept; however, published studies about the situation in Italy were lacking. This article contributes to the existing evidence, confirming these associations in the Italian context, and provides new data that support a more holistic approach in public health interventions: mental and physical health should be considered as a combined entity, and health promotion strategies should emphasize prevention by tackling modifiable physical and mental health risk factors with concurrent and integrated interventions.
